# Case Report: Antenatal diagnosis of double-chambered right ventricle in two cases: follow-up and literature review

**DOI:** 10.3389/fcvm.2026.1760854

**Published:** 2026-04-22

**Authors:** Kazim Oztarhan, Aysin Kale, Idil Kacur, Aylin Oztarhan, Selda Atar, Ali Gedikbasi

**Affiliations:** 1 University Hospital Division of Pediatric Cardiology, Istanbul Medical School, İstanbul, Türkiye; 2 Division of Anatomy, Istanbul Medical School, İstanbul, Türkiye; 3 Department of Obstetrics and Gynecology, Health Science University Istanbul Sisli Etfal Hospital, İstanbul, Türkiye; 4 Beylikduzu Surgery Center, Department of Obstetrics and Gynecology, Acibadem Hospitals, İstanbul, Türkiye; 5 Private Clinic, Prenatal Center, İstanbul, Türkiye

**Keywords:** double chamber right ventricle (DCRV), hemodynamic obstruction and criteria, prenatal diagnosis, type 1 DCRV, type 2 DCRV

## Abstract

Double-chambered right ventricle (DCRV) is defined as the progressive division of the right ventricle into two chambers: a high-pressure inlet chamber and a low-pressure outlet chamber. To date, only three cases diagnosed prenatally have been reported in the literature, all of which were associated with unfavorable pregnancy outcomes. In this study, we discuss DCRV in general, along with two cases of type 1 DCRV that did not cause hemodynamically significant obstruction and resulted in successful pregnancy outcomes. From a hemodynamic perspective, the following echocardiographic criteria may affect the prenatal fetal process: (1) detection of tricuspid regurgitation on echocardiography; and (2) pulmonary blood flow velocity and the difference in pressure between the proximal and distal right ventricle.

## Introduction

Double-chambered right ventricle [DCRV, also known as right ventricular anomalous muscle bundle (RVAMB)], is a congenital heart defect of the right ventricle (RV), detected in 1 in 36,000 autopsies (1). First described by Peacock in 1858 as a mid-cavitary right ventricular outflow tract obstruction, DCRV is defined as a progressive division of the RV into two chambers: a high-pressure inlet chamber and a low-pressure outlet chamber (1–4).

Although many publications and case reports on DCRV in children and adults exist, only three cases with an antenatal diagnosis have been reported in the literature to date (5–7). In two of these cases, delivery was performed urgently and mandatorily at 28 weeks of gestation due to hydrops in one and heart failure in the other; both neonates subsequently died postnatally (6, 7). In the remaining surviving case, cardiac congestive failure developed at 3 months of age; surgery was performed at 6 months after left ventricular end-diastolic measurements were found to be enlarged, along with increased ventricular and pulmonary artery pressures (5). In 2011, Park et al. evaluated DCRV from a hemodynamic perspective and classified it into two types (8). These published prenatal cases were of the obstructive form and can be classified as type 1 DCRV according to the Park classification.

In this study, we discuss two cases of type 1 DCRV that do not cause hemodynamically significant obstruction, according to Park's classification. In addition, we provide data on hemodynamic factors that may affect the fetal process.

## Case report

### Case 1

**Figure 1:**
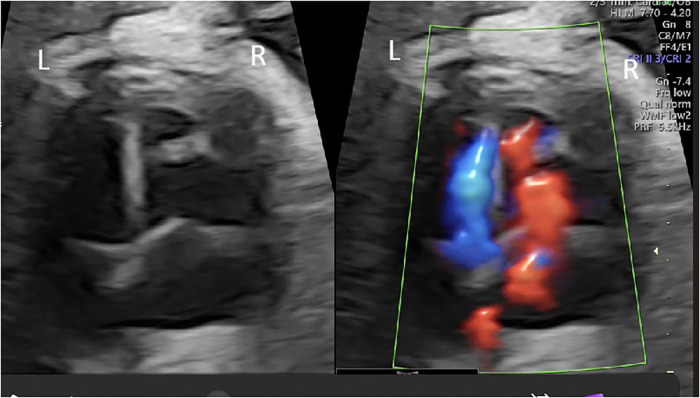
Simultaneous B-mode and color Doppler ultrasound image of the first case. In addition to the mitral and tricuspid valves, a more remarkable structural formation within the right ventricle and inflow toward the most distal region can be seen with color Doppler.

A 24-year-old woman, gravida 1 para 0, was referred to our Department of Maternal Fetal Medicine at 24 weeks of gestation for detailed sonographic evaluation. Detailed echocardiography revealed a congenital cardiac anomaly consistent with DCRV. The semilunar valves and the tricuspid valve inlet appeared normal. The right ventricle was partially divided by an extremely hypertrophic muscular band into a superobasal inflow sac and a narrower inferoapical sac (Figure 1, Supplementary Video S1), resulting in inflow narrowing. Color Doppler flow measurement showed minimal reverse flow across the tricuspid valve, originating from the communicating channel (opening between the upper and lower parts) of the divided right ventricle. The pulmonary valve was normally positioned, well above the hypertrophied band, with normal flow velocity and normal time to peak velocity. The umbilical artery and the middle cerebral artery also showed normal flow patterns. The family received relevant counseling, including information from the relevant literature. Amniocentesis with comparative genomic microarray analysis (CMA) were offered and performed. Despite the unfavorable outcomes reported in the previously published prenatal DCRV cases, the decision was made to continue the pregnancy with close follow-up, including assessments at every 2 weeks and special attention at 28 weeks of gestation. Meanwhile, both karyotype and CMA results were normal. The patient was followed up uneventfully until the 38th gestational week, and delivery was performed by cesarean section at 38+ gestational week at the request of the patient.

A 4,030-g female infant was delivered without complications, with Apgar scores of 7, 9, and 10 at 1, 5, and 10 min, respectively. Postnatal cross-sectional echocardiography during the first week of life confirmed the prenatal diagnosis of type 1 DCRV (Supplementary Video S1). Repeated echocardiography at 8 months of age showed persistent findings without any adverse clinical outcomes.

### Case 2

A 32-year-old pregnant woman, gravida 1 para 0, was referred for detailed sonographic evaluation at 21 weeks of gestation. Detailed echocardiography showed abnormal RV development consistent with DCRV, with normal semilunar valves. The tricuspid valve appeared normal; however, in addition to the moderator band, an apically located hypertrophic muscle band was identified within the right ventricle. Color Doppler flow measurement showed minimal flow through the communicating channel of the divided right ventricle (between the proximal and distal portions). The pulmonary valve was normally positioned, well above the hypertrophied band, with normal flow measurements. Other fetal Doppler measurements were within normal limits (Supplementary Video S2). Amniocentesis was recommended to evaluate for possible chromosomal abnormalities; however, the family declined the invasive procedure.

Although regular fortnightly pregnancy and echocardiographic follow-up was recommended, the family's visits were irregular. At the 39th gestational week, a 3,990-g female infant was delivered by cesarean section due to breech presentation in a primigravida by her own physician, with APGAR scores of 6, 9, and 10 at 1, 5, and 10 min, respectively. Postnatal cross-sectional echocardiography performed during the first week of life confirmed the prenatal diagnosis of type 1DCRV. Follow-up echocardiography at 9 months of age showed persistent findings without any adverse clinical outcomes (Supplementary Video S2).

## Discussion

DCRV probably occurs due to variation in the elevations within the right ventricle, leading to its division into a proximal high-pressure chamber and a distal low-pressure chamber by a huge ridge that obstructs blood flow (1, 3, 9). In most DCRV cases, this ridge is formed by a muscle bundle traversing the right ventricle, typically located inferior to the infundibulum. In some cases, multiple muscle bundles may be present rather than a single structure (9). The terms “anomalous right ventricular muscle bundle” and “double-chambered right ventricle” are used interchangeably, reflecting the variety of lesions that can cause partitioning of the RV. Restivo et al. (10) reported seven different subtypes of double-chambered right ventricle based on abnormal muscular findings observed at necropsy.

DCRV can be classified into two types; in the first type, an abnormal muscle bundle traverses the right ventricle, whereas in the second type, there is parietal or septal muscle hypertrophy (11). Park et al. further categorized these as type 1 DCRV, due to the presence of abnormal muscle tissue crossing the RV, and type 2 DCRV, associated with parietal and septal muscle hypertrophy (8). A defect is defined as “high” when located close to the pulmonary valve and “low” when localized in the apex. “Diffuse” defects are triangular with long attachments, where “discrete” defects are well-defined and have short attachments (12).

From a topographical perspective, the right ventricle consists of three components: the inlet, the trabeculated apical part, and the muscular outlet. The inlet surrounds the tricuspid valve, the trabeculated apical part is located near the cardiac apex, and the outlet lies around the pulmonary valve, with its surrounding structures supporting the cusps (11). The inlet and outlet components are located at the roof of the right ventricle and are separated from by the supraventricular crest. This crest comprises a parietal component (the ventriculoinfindibular fold) and a septal component (the outlet or infindibular septum). The ventriculoinfundibular fold separates the tricuspid and pulmonary valves, while the infindibular septum separates the pulmonary and aortic outlets (3). The supraventricular crest itself is formed by muscular tissue.

Two key morphological features of the RV are the supraventricular crest and the septomarginal trabeculation. The supraventricular crest is a muscular shelf that separates the tricuspid and pulmonary valves. It is, in fact, an infolding of the inner curvature of the heart, referred to as the ventriculoinfindibular fold. This fold inserts into the septomarginal trabeculation, which is located at the apex of the ventricle. The septomarginal trabeculation is a Y-shaped band that is continuous with the interventricular septum at the base of the heart and gives rise to two limbs: an anterosuperior (septal) limb and a posteroinferior (parietal) limb. The septal limb extends superiorly to cover the outlet part of the muscular ventricular septum and supports the pulmonary valve within the outlet of the ventricle. The parietal limb extends posteroinferiorly toward the interventricular component of the membranous septum, providing support for this muscular structure (3). The body of the septomarginal trabeculation continues toward the apex of the RV, where it splits into several smaller trabeculations known as septoparietal bands. Among these, one trabeculation is very prominent: the moderator band. This band attaches to the apex and base of the interventricular septum and then connects to support the anterior papillary muscle before crossing over to the free wall of the ventricle. Some septoparietal bands may vary in size and can appear large enough to be mistaken for the moderator band (3).

**Figure 2:**
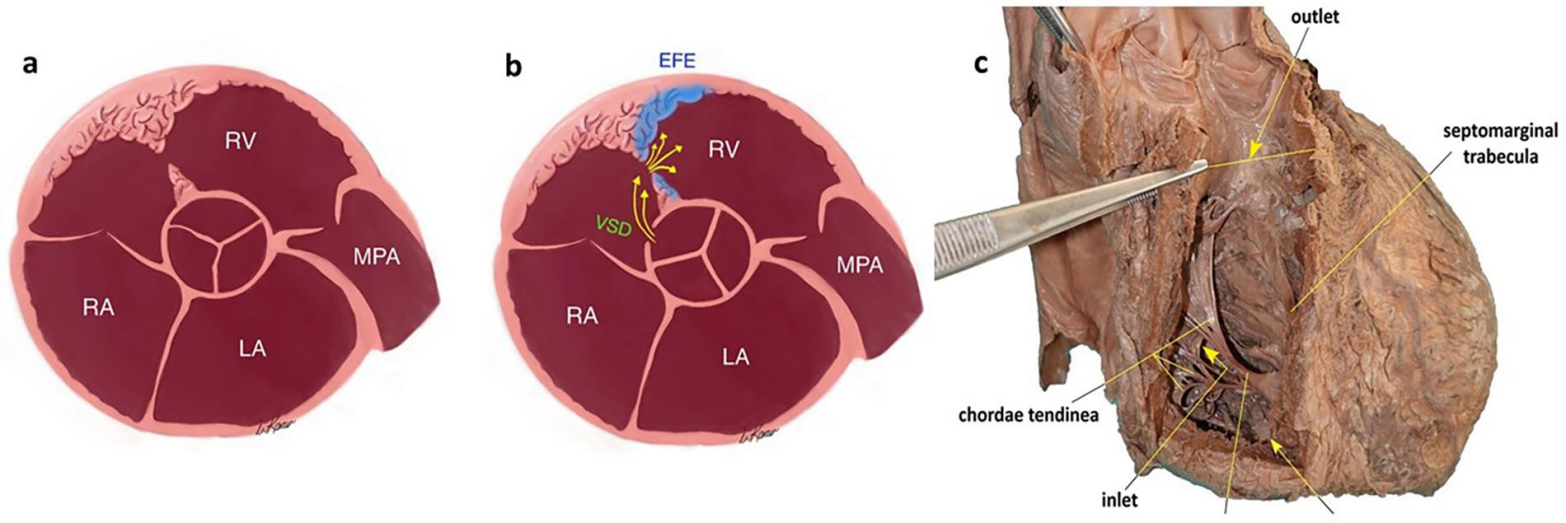
**(a)** Type 1 DCRV: developmental mechanism involving abnormally positioned muscular bands, hypertrophy or displacement of the moderator band, and abnormal hypertrophy of the right ventricular trabecular musculature. **(b)** Type 2 DCRV: increased blood flow and pressure within the right ventricle (via VSD), leading to the development of abnormal muscle structures. LA, left atrium; RA, right atrium; RV, right ventricle; MPA, main pulmonary artery; VSD, ventricular septal defect; EFE, endocardial fibroelastosis. **(c)** Normal anatomy of the right ventricle.

The most common developmental mechanism of DCRV involves congenital abnormalities of the muscular structures. These include abnormally positioned muscle bands, hypertrophy or displacement of the moderator band, and abnormal hypertrophy of the right ventricular trabecular musculature. Thickening of these structural elements, , particularly within the supraventricular crest, may eventually lead to the formation of chambers within the right ventricle (2) (Figure 2). Whether primary DCRV has a genetic basis will become apparent as more cases are reported.

In addition, *de novo* cases of DCRV have been reported. Folger (13) described the development of newly emerging intraventricular pressure gradients associated with the formation of a contractile pouch in the outflow tract. These findings suggest that postnatal flow patterns within the RV may cause hypertrophic changes in the trabeculae. According to Park, type 2 DCRV develops secondary to congenital heart disease, where increased blood flow and pressure within the right ventricle lead to the formation of abnormal muscle structures. As explained by Nikolic et al. (14), DCRV progression is associated with left-to-right shunting through a VSD, which generates a jet directed toward the RV free wall or crista supraventricularis, suggesting that flow turbulence contributes to progressive obstruction. In all patients, flow abnormalities were observed along abnormal muscle bundles associated with RVOT obstruction and VSD. Histological analysis of tissue resected from the RVOT during surgical repair of DCRV revealed that myocardial hypertrophy alone does not account for this septation formation; instead, the resected tissue resembled fibroelastic tissue, with distinct layers of collagen and elastic fibers evidence of infiltrative growth into the underlying myocardium. DCRV develops not only as a result of hypertrophy of muscle bundles but also through an invasive remodeling process involving the endocardium and the underlying myocardium (15) (Figure 2).

In terms of prognosis, the location of these band formations and the degree of obstruction they produce are important adverse prognostic signs: significant narrowing can reduce pulmonary blood flow, leading to increased tricuspid insufficiency, right-sided heart failure, and the development of hydrops, ultimately resulting in serious intrauterine complications. Therefore, the short-axis view for evaluating the right ventricular outflow tract is very important in both antenatal and postnatal follow-up (Figure 2, Supplementary Video S2). This may explain the two reported cases (6, 7) of hydrops and cardiac failure. The case reported by Leandro et al. (5) involved a lesion located closer to the pulmonary valve (at the infundibular level) and was associated with severe obstruction and significant tricuspid insufficiency. From this perspective, hemodynamically significant and non-significant DCRV cases can be assessed based on the following criteria: (1) the presence of tricuspid regurgitation on echocardiography; (2) pulmonary flow velocity and the pressure gradient between the proximal and distal portions of the right ventricle. In particular, as the pressure gradient between the proximal and distal right ventricles increases—reflecting worsening obstruction—tricuspid insufficiency becomes more pronounced. Since the right ventricle is the dominant ventricle in the fetus, progressive obstruction may lead to heart failure and, in later stages, to fetal hydrops (3). In addition, in DCRV, hypoplasia of the distal chamber in later gestational weeks, combined with reduced pulmonary blood flow velocity, may eventually lead to the development of pulmonary hypoplasia. Therefore, in cases of severe obstruction in DCRV, monthly follow-up is recommended during the perinatal period.

Invasive genetic testing may be performed when requested by the family or in the presence of additional high-risk findings (such as anomalies in other organ systems or a family history of genetic disorders).

In both of our cases, the lesions were located low, near the apex, and had no or minimal impact on pulmonary stroke volume. The absence of additional pathology was another factor that favorably affected the prognosis in our cases.

Although we reported no additional malformations during pre- and postnatal sonographic evaluation, there is a high incidence of coincident congenital disease in DCRV cases; up to 62%–90% of cases are associated with membranous-type ventricular septal defects (3, 4, 8). Other coexisting abnormalities are predominantly right-sided pathologies, including pulmonary valve stenosis, pulmonary atresia, tricuspid valve regurgitation, double-outlet RV, Ebstein's anomaly, persistent left superior vena cava, ruptured sinus of Valsalva aneurysm, and other cardiac malformations like anomalous pulmonary drainage, transposition of the great arteries, atrial septal defect, aortic valve regurgitation, and tetralogy of Fallot (3, 4, 8, 13). In addition, arrhythmias are a common additional complication in children with DCRV, especially after surgery (3). The tendency of DCRV to occur in association with other congenital cardiac abnormalities, and its rare occurrence as an isolated condition, suggests a strong developmental correlation (8).

Isolated double-chambered right ventricle (DCRV) may remain asymptomatic for years and typically presents later in life due to progressive intraventricular obstruction. The resulting increased hemodynamic burden can manifest as a systolic murmur, chest pain, palpitations, or heart failure. As a rare entity, it is often diagnosed incidentally at an advanced age (8).

However, a two-case report does not provide sufficient evidence to support a formal classification recommendation.

This two-case series does not provide sufficient evidence to support a formal classification proposal. However, we present this as a hypothesis to support the understanding of the pathophysiological development of DCRV. Future studies with larger cohorts are needed to validate this proposed framework. Nevertheless, the development of obstruction during pregnancy, regardless of the pathophysiological type, warrants particularly careful monitoring.

The scarcity of published literature on fetal cases is likely attributable to the rarity of DCRV, difficulties in identifying mild forms, and the loss of severe obstructive cases during early pregnancy due to severe heart failure. It should be noted that f non-obstructive type 1 DCRV detected during the prenatal period may represent a stable anatomical variant and, in most cases, can be safely managed with serial echocardiographic follow-up rather than urgent intervention. Cases reported in studies other than ours typically present with early and severe findings, prompting detailed evaluation and, consequently, resulting in loss to follow-up during pregnancy. In cases where the condition is mild, it is often easily overlooked during fetal evaluation.

A comparative evaluation of the two cases highlighted common features associated with a favorable prognosis in both patients. These include (1) absence of significant obstruction in the right ventricular outflow tract; (2) preserved pulmonary artery flow; and (3) absence of additional structural cardiac anomalies.

Accordingly, a long-term follow-up plan has been established for these patients. The follow-up program is planned as follows: (1) pediatric cardiology evaluation with transthoracic echocardiography every 6 months during the first 2 years; (2) annual cardiology follow-up thereafter, provided clinical findings remain stable; and (3) echocardiographic evaluation focusing on (a) the morphology of the muscular bands within the right ventricle; (b) right ventricular right ventricular outflow tract velocity; (c) the presence and degree of tricuspid regurgitation; and (d) the pressure gradient between the proximal right ventricle and the pulmonary artery.

In cases of type 1 DCRV diagnosed during the fetal period, those with hemodynamically insignificant findings can be safely managed with regular follow-up. Invasive genetic testing is recommended only in high-risk situations, and surgical intervention should be considered only if severe obstruction or clinical symptoms develop.

As many pathologies can affect intraventricular pressure dynamics, it is important to differentiate DCRV from other forms of right ventricular outflow tract (RVOT) obstruction. DCRV is the only RVOT obstruction classified as subinfundibular; other reasons of RVOT obstruction occur either at or above the valve, at the infundibulum (2, 8).

Transthoracic echocardiography is typically sufficient for diagnosis in pediatric patients. However, in adults, a transesophageal approach provides better visualization of congenital lesions (8). Bashore (16) stated that transesophageal echocardiography is not necessary for the diagnosis of DCRVand that magnetic resonance imaging (MRI) may be the best imaging modality. Although echocardiography is diagnostic in double-chambered ventricles (left and right ventricles), it is accepted that it may occasionally fail to detect abnormal muscular bands and that MRI can be useful for anatomical assessment a definitive diagnosis (8, 16, 17).

Indications for surgery may include a pressure gradient exceeding 40 mmHg between the proximal chamber and the pulmonary artery, the presence of aortic regurgitation, and symptoms of heart failure (3, 18). The most effective treatment for DCRV is the surgical removal of pathological tissue via right atriotomy or right ventriculotomy (3, 4, 18). Both children continue to be followed-up, with no adverse findings.

Due to the rarity of primary DCRV presenting with obstruction and hydrops fetalis during fetal life, studies on this condition are very limited. Our aim is to highlight the possibility that the primary non-obstructive form may also be seen during the early stages. Key echocardiographic findings include changes in right ventricular pressure, detected as tricuspid insufficiency, and changes in pulmonary artery blood flow. The right ventricular chamber should be carefully assessed in both the apical four-chamber view and the parasternal short-axis view, using both B-mode and Doppler mode to make timely obstetric decisions.

## Conclusion

This study highlights that prenatally diagnosed type 1 DCRV without significant hemodynamic obstruction may represent a stable anatomical variant in selected cases. A favorable prognosis is associated with the absence of RVOT obstruction, preserved pulmonary blood flow, and the absence of additional cardiac anomalies. Accurate prenatal diagnosis requires detailed morphological and Doppler evaluation, and regular prenatal and postnatal follow-up remains essential. Further large-scale and long-term studies are needed to clarify the natural history and refine the classification of DCRV.

## Data Availability

The original contributions presented in the study are included in the article/Supplementary Material, further inquiries can be directed to the corresponding author.

## Supplementary material

Video 1 First case of DCRV at 24 weeks of gestation, follow-up videos during pregnancy, and postnatal echocardiography images.

Video 2 Second case of DCRV diagnosed at 21 weeks of gestation, with follow-up videos during pregnancy and postnatal echocardiography images.
